# Clinical utility of arterial spin labeling for preoperative grading of glioma

**DOI:** 10.1042/BSR20180507

**Published:** 2018-08-31

**Authors:** Jun Fu, Linchen Li, Xinjun Wang, Min Zhang, Yan Zhang, Zhanzhan Li

**Affiliations:** 1 Department of Oncology, Xiangya Hospital, Central South University, Changsha, Hunan Province 410008, China; 2 Department of Neurosurgery, The Fifth Affiliated Hospital of Zhengzhou University, Zhengzhou, Henan Province 450000, China; 3 Department of Pathology, The Second Affiliated Hospital of Zhengzhou University, Zhengzhou, Henan Province 450014, China

**Keywords:** arterial spin labeling, diagnostic value, Glioma, meta-analysis

## Abstract

There were obvious differences in biological behavior and prognosis between low- and high-grade gliomas, it is of great importance for clinicians to make a right judgement for preoperative grading. We conducted a comprehensive meta-analysis to evaluate the clinical utility of arterial spin labeling for preoperative grading. We searched the PubMed, Embase, China National Knowledge Infrastructure, and Weipu electronic databases for articles published through 10 November 2017 and used ‘arterial spin-labeling’ or ‘ASL perfusion, grading’ or ‘differentiation, glioma’ or ‘glial tumor, diagnostic test’ as the search terms. A manual search of relevant original and review articles was performed to identify additional studies. The meta-analysis included nine studies. No obvious heterogeneity was found in the data in a fixed-effect model. The pooled sensitivity and specificity were 90% (95% confidence interval (CI): 0.84–0.94) and 91% (95% CI: 0.83–0.96), respectively, and the pooled positive likelihood ratio (PLR) and negative likelihood ratio (NLR) were 10.40 (95% CI: 2.21–20.77) and 0.11 (95% CI: 0.07–0.18). The diagnostic odds ratio (DOR) was 92.47 (95% CI: 39.61–215.92). The diagnostic score was 4.53 (95% CI: 3.68–5.38). The area under the curve (AUC) was 0.94 (95% CI: 0.91–0.96). Subgroup analyses did not change the pooled results. No publication bias was found (*P*=0.102). The normalized maximal tumor blood flow/normal white matter ratio obtained with the arterial spin labeling technique was relatively accurate for distinguishing high/low-grade glioma. As a non-invasive procedure with favorable repeatability, this index may be useful for clinical diagnostics.

## Introduction

A glioma is a neuroepithelial tumor with glial cell phenotypic characteristics. Gliomas are the most common primary intracranial tumors in adults [1] and account for ~30% of all central nervous system tumors and 80% of malignant brain tumors [2]. The incidence of glioma is increasing gradually amongst the elderly population [3]. Because of the large differences in biological behavior and prognosis between low- and high-grade gliomas, it is important that clinicians accurately assess the preoperative grade, as this is in turn important when selecting the surgery type and for predicting the treatment outcome [4,5]. Magnetic resonance perfusion imaging is one of the most common method used for tumor grading [6,7]. Arterial spin labeling, which uses water molecules as an endogenous tracer, is a non-invasive procedure with favorable repeatability [8]. This technology has been drawing increasing attention in clinical practice. Several studies have reported on the application of arterial spin labeling to glioma grading [9–12]. Individual studies have some limitations because of small sample sizes and the pathology pattern, and thus cannot fully delineate the diagnostic value of arterial spin labeling. Until now, no relevant meta-analysis has been reported. Therefore, we conducted a comprehensive analysis to evaluate the clinical utility of arterial spin labeling for preoperative glioma grading using the normalized tumor blood flow (nTBF; maximal tumor blood flow/normal white matter). Our results provide important diagnostic guidance for clinical practice.

## Materials and methods

### Search strategy

We conducted this meta-analysis based on an observational epidemiological protocol (Preferred Reporting Items for Systematic Reviews and Meta-Analyses Checklist, Research Checklist). We searched the PubMed, Embase, Web of Science, China National Knowledge Infrastructure, Wanfang, and Weipu electronic databases for articles published through 30 April 2018 and used ‘arterial spin-labeling’ or ‘ASL perfusion, grading’ or ‘differentiation, glioma’ or ‘glial tumor, diagnostic test’ as the search terms. A manual search of relevant original and review articles was performed to identify additional studies. Our inclusion criteria were based on the study topic, design, participant characteristics, exposure, control, and reported outcomes. We restricted the language of publication to Chinese and English. The search strategy is presented in Supplementary material.

### Selection criteria

All included studies met the following criteria: (i) differentiation between low- and high-grade gliomas using arterial spin labeling; (ii) all patients confirmed pathologically; (iii) patients did not receive chemoradiotherapy; (iv) inclusion of data on the true-positive (TP), false-positive (FP), false-negative (FN), and true-negative (TN) rates; and (v) published in English or Chinese. Duplicates, reviews, comments, and animal experiments were excluded.

### Data extraction and assessment of quality

For each study, the following information was extracted: author, year of publication, sample size, age, male/female ratio, tumor stage (low or high), machine type, and method. Two investigators extracted the data independently and disputes were settled by negotiation. We used the updated Quality Assessment of Diagnostic Accuracy Studies 2 (QUADAS-2) to assess the quality of the selected studies [13]. This tool comprises four main parts: patient selection, index test, reference standard and patient flow, and timing of the index tests, and reference standard. The signaling questions were answered as ‘yes’, ‘no’, or ‘unclear’, and are phrased such that ‘no’ denoted a high risk of bias, ‘yes’ indicated a low risk of bias, and ‘unclear’ indicated an unclear risk of bias.

### Statistical analysis

The heterogeneity of the threshold effect was evaluated using the Spearman test [14]. The heterogeneity amongst studies was assessed using Cochran’s *Q* and the *I^2^* statistic; *I^2^* > 50% and *P*<0.05, respectively, indicated significant heterogeneity [15,16]. The following data were pooled: sensitivity, specificity, positive likelihood ratio (PLR), negative likelihood ratio (NLR), diagnostic odds ratio (DOR), and the summary area under the curve (AUC) (and their confidence intervals; CI). An AUC of 1.0 means perfect diagnostic ability, while an AUC close to 0.5 indicates poor diagnostic ability. We used a linear regression analysis to examine publication bias [17]. Subgroup analyses were conducted according to machine type and method. All analyses were conducted using Stata 14.0 (StataCorp LP, College Station, TX, U.S.A.). *P*<0.05 indicated statistical significance.

## Results

### Literature search

**Figure 1:**
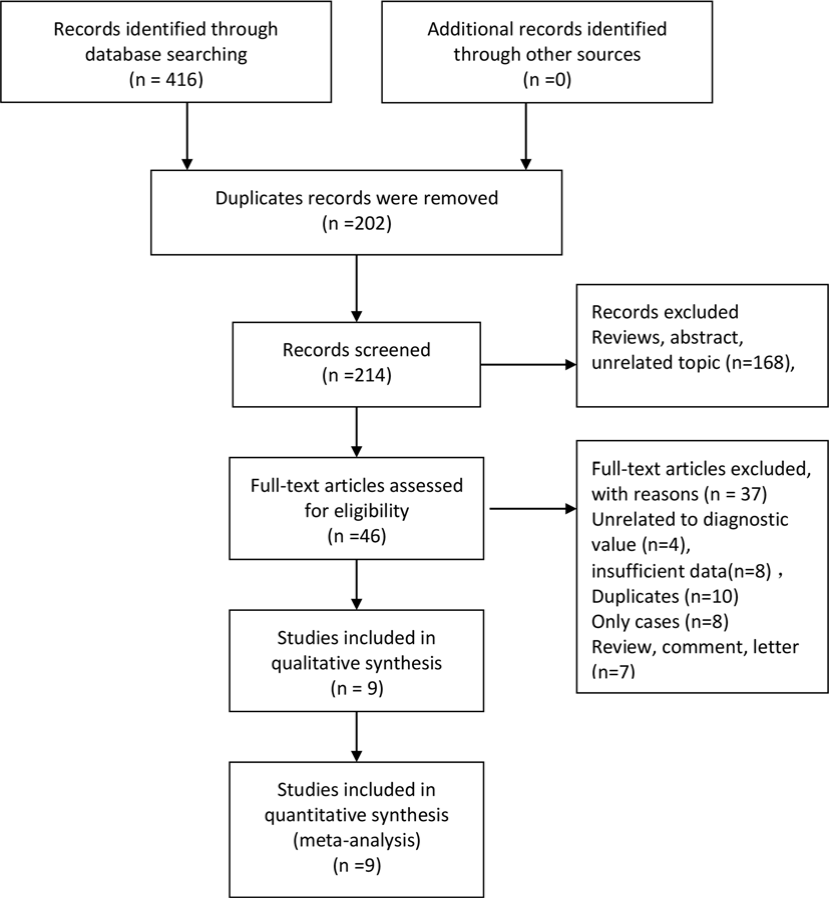
Flow diagram of studies selection

Figure 1 outlines the study selection process. We obtained 416 records in the initial database search, from which 202 duplicates were removed. After screening, 168 records were excluded for different reasons and 46 potentially eligible studies were identified. After scanning the full text, 37 studies were excluded for the following reasons: unrelated to diagnostic value (*n*=4), insufficient data (*n*=8), duplicates (*n*=10), cases only (*n*=8), and reviews, comments, or letters (*n*=7). Ultimately, the meta-analysis included nine studies [11,12,18–24]. A manual search of the references listed in the following studies did not return any additional potential studies.

### Study characteristics and quality assessment

Table 1 shows the general characteristics of the included studies. The nine studies included 339 glioma patients (142 low-grade and 197 high-grade gliomas). The following studies were published from 2011 to 2016. The mean and median patient ages were more than 40 years, and there were 182 males and 157 females. All studies were conducted in the Asian-Pacific region. One study used a Siemens 3.0 T scanner, seven used a GE 3.0 T, and one used a GE 1.5 T. Five studies used 3D pseudo-continuous arterial spin labeling and four used the pulsed arterial spin labeling (PASL) method. All patients and glioma grades were confirmed pathologically. All the included studies received moderately high scores in the QUADAS-2 quality assessment. The included studies were relatively good in terms of patient selection, index test, reference standard, and patient flow and timing of the index tests, and reference standard. The quality assessment is presented in Supplementary Figures S1 and S2.

**Table 1:** General characteristics of included studies in the meta-analysis

Author	Year of publication	Sample size	Age	Male/female	Tumor stage (low/high)	Machine	Methods	Gold standard
Wang	2011	31	42.9	16/15	12/19	Seimens 3.0T	PASL	Pathology
Qiao	2015	28	50	21/7	11/17	GE 3.0T	3D PCASL	Pathology
Wang	2016	37	41	–	14/23	GE 3.0T	3D PCASL	Pathology
Liao	2016	41	47	23/18	20/21	GE 3.0T	3D PCASL	Pathology
Tian	2015	45	40.3	25/20	19/26	GE 3.0T	3D PCASL	Pathology
Zheng	2014	21	42.7	13/8	5/16	GE 3.0T	PASL	Pathology
Jiang	2014	23	54	12/11	10/13	GE 3.0T	PASL	Pathology
Kim	2008	61	43	26/32	26/35	GE 1.5T	PASL	Pathology
Shen	2016	52	–	–	25/27	GE 3.0T	3D PCASL	Pathology

Abbreviation: PCASL, pulsed continuous arterial spin labeling

### Diagnostic accuracy: pooled results

**Figure 2:**
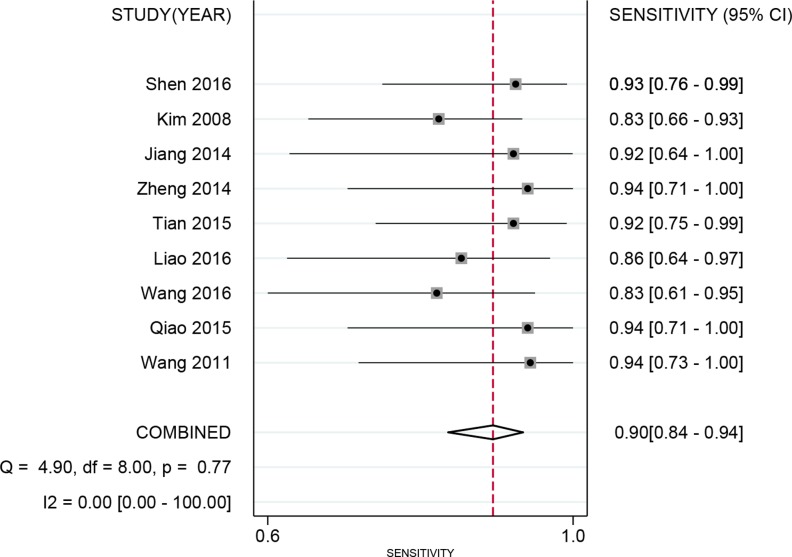
Forest plot of pooled sensitivity of arterial spin labeling for preoperative grading of glioma

**Figure 3:**
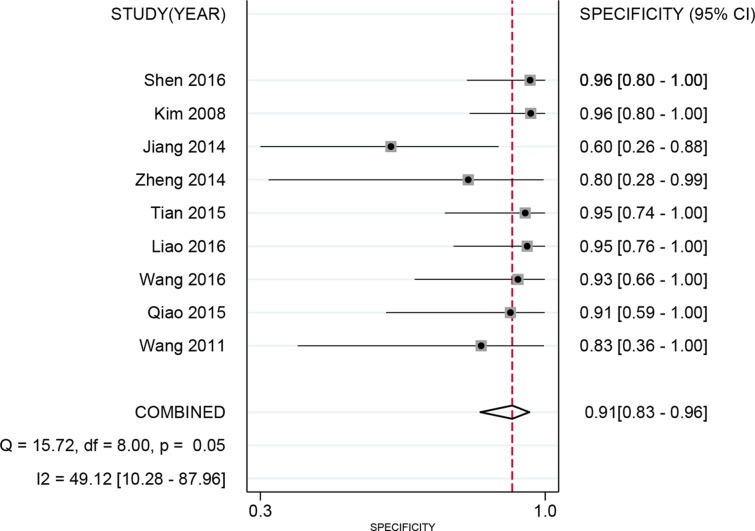
Forest plot of pooled specificity of arterial spin labeling for preoperative grading of glioma

**Figure 4:**
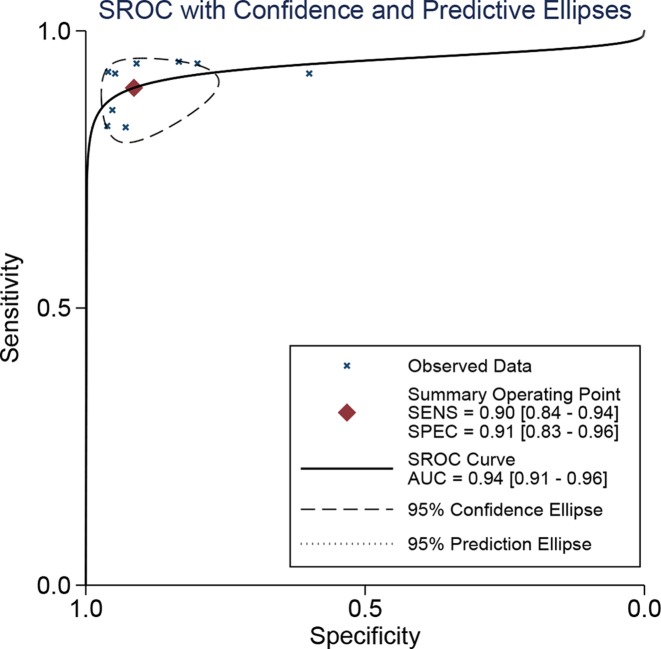
The symmetric receiver operating characteristic curve of arterial spin labeling for preoperative grading of glioma

**Figure 5:**
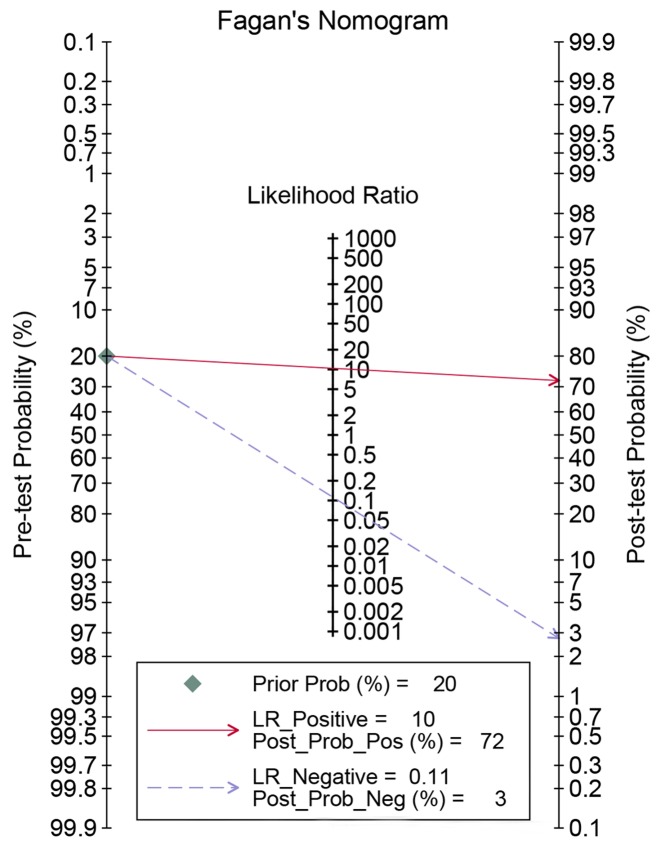
Fagan diagram evaluating the overall diagnostic value arterial spin labeling for preoperative grading of glioma (if the pretest probability is 20% for a patient, the post-test probability will be 72% with a PLR of 10)

Spearman’s correlation showed that there was no heterogeneity caused by a threshold effect (*r* = 0.496, *P*=0.175). Cochran’s *Q* showed no obvious heterogeneity in the meta-analysis data in a fixed-effect model. The pooled sensitivity and specificity were 90% (95% CI: 0.84–0.94, Figure 2) and 91% (95% CI: 0.83–0.96, Figure 3), respectively. The *I^2^* of heterogeneity for the sensitivity and specificity was 0.0 and 49.1% (*P*=0.770 and *P*=0.050, respectively). The pooled PLR and NLR were 10.40 (95% CI: 2.21–20.77) and 0.11 (95% CI: 0.07–0.18), respectively. The DOR was 92.47 (95% CI: 39.61–215.92). The diagnostic score was 4.53 (95% CI: 3.68–5.38). Figure 4 shows the summary receiver operating characteristic (SROC) curve for nTBF (AUC = 0.94, 95% CI: 0.91–0.96, Figure 4). Figure 5 shows Fagan’s nomogram and indicates that the post-test probability was 72% if the pre-test probability was 20%. Subgroup analyses were conducted according to the machine type and method. For machines, the pooled sensitivity was 89% (95% CI: 83–93%) and the specificity was 92% (95% CI: 83–96%). The pooled PLR and NLR were 11.01 (95% CI: 5.19–23.32) and 0.12 (95% CI: 0.08–0.19), respectively. The DOR was 37.09 (95% CI: 21.37–64.36). The pooled AUC was 0.93 (95% CI: 0.91–0.95). For 3D pulsed continuous arterial spin labeling (PCASL), the sensitivity, specificity, PLR, NLR, and DOR were 90% (95% CI: 82–94%), 94% (95% CI: 87–98%), 16.11 (95% CI: 6.86–37.84), 0.11 (95% CI: 0.07–0.19), and 144.50 (95% CI: 48.96–426.46), respectively. The AUC was 0.97 (95% CI: 0.95–0.98). For PASL, the sensitivity, specificity, PLR, NLR, and DOR were 91% (95% CI: 80–96%), 84% (95% CI: 60–95%), 5.66 (95% CI: 2.06–15.53), 0.11 (95% CI: 0.05–0.25), and 51.41 (95% CI: 14.20–186.08), respectively. The AUC was 0.94 (95% CI: 0.92–0.96).

### Publication bias

**Figure 6:**
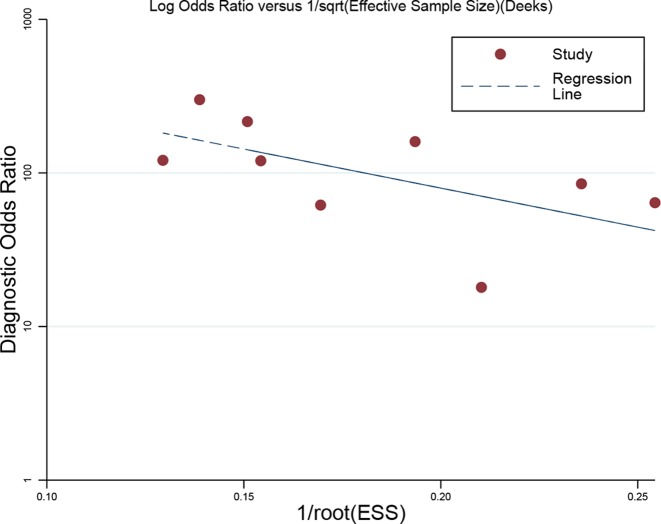
Line regression plot of publication bias

**Figure 7:**
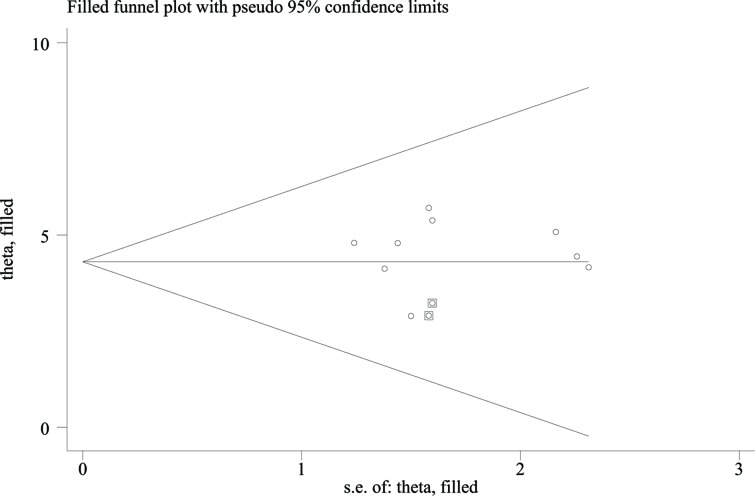
Filled funnel plot of publication bias

We used linear regression to evaluate the publication bias. As shown in Figure 6, the *P*-value of the slope coefficient for publication bias was 0.102. We also used the trim and fill method to assess the publication bias. The trim and fill method has been presented in Figure 7. According to the results, only two studies were needed for symmetry of funnel plot. No significant difference was observed for pooled effect size (before trim and filled: fixed: 4.58 (95% CI: 3.53–5.63); random: 4.58 (95% CI: 3.53–5.63)), and after filled: fixed: 4.30 (95% CI: 3.53–5.25); random: 4.30 (95% CI: 3.53–5.25). No publication bias was found and our results were stable.

## Discussion

Our results indicated that the normalized maximal tumor blood flow/normal white matter ratio determined using arterial spin labeling aids in the pathology grading of gliomas (AUC = 0.94). The nTBF showed high sensitivity (90%) and specificity (91%). These results indicate that nTBF is a useful diagnostic index for grading gliomas, especially as high or low grade.

The treatment strategy and clinical prognosis differ markedly amongst different grades of glioma. Therefore, accurate assessment of the grade of glioma is important for the treatment plan, which may include surgery, postoperative radiotherapy, and chemotherapy [25]. Routine examination of asymptomatic patients includes computed tomography (CT) and MRI. Head CT is used to determine whether there is an initial intracranial space and MRI shows the characteristics and properties of a glioma better than CT. Consequently, MRI has become the preferred method for evaluating gliomas. However, preoperative grading of a glioma often relies only on conventional MRI, which is frequently insufficient [26]. The degree of malignancy of a tumor is closely related to the microvessel structure and tumor cell proliferation. Therefore, it is important to evaluate angiogenesis in a glioma to determine the degree of malignancy. Because gliomas have rich vascularity, their vascular structure differs significantly from that of normal brain tissue, in terms of both the intrinsic cerebrovascular structure and neovascularization. The new blood vessels of a glioma are characterized by high density, severe distortion, and non-uniform vascular diameter. Therefore, the degree of neovascularization is an important indicator of the glioma grade, which provides a theoretical basis for using perfusion-weighted imaging (PWI) for the diagnosis and classification of gliomas. Current perfusion techniques using tracer contrast agents proceed as follows: (i) intravenous rapid injection of an exogenous contrast agent, T1-weighted dynamic contrast-enhanced MRI (DCE-MRI), and evaluation of the effects of lateral relaxation on T2*/T2-weighted dynamic susceptibility contrast PWI (DSC-PWI); and (ii) the use of protons in water as an endogenous tracer [27,28]. DSC-PWI is now the most commonly applied MRI perfusion technique. In recent years, progress has been made with respect to application of DSC-PWI to the pathological grading of gliomas. However, DSC-PWI and DCE-MRI require high-pressure injection of an exogenous contrast agent, and the technician must determine the scanning time accurately. Therefore, their use is limited in patients with severe renal insufficiency or allergies to contrast medium, and in those who cannot co-operate with the examination. High-pressure syringes and contrast medium are expensive and repeated examinations can be an economic burden for patients [29].

As a non-invasive perfusion technique, arterial spin labeling is more sensitive than traditional DSC-PWI. Arterial spin labeling can be used to evaluate the microvascular distribution and tumor blood perfusion, both qualitatively and quantitatively. Moreover, arterial spin labeling is not affected by the blood–brain barrier, and truly reflects the degree of perfusion. Arterial spin labeling can be applied repeatedly in children and restless patients [30] and assesses cerebral blood flow, which represents blood flow in a specific organ per unit. Tumor angiogenesis usually occurs during tumor progression from low to high grade and is characterized by increased blood flow. Although tumor blood flow is useful for grading gliomas, this index is affected by age and patient factors. Compared with direct tumor blood flow, the blood flow/normal blood flow ratio and nTBF enable more accurate assessment [31]. The nTBF consists of three indexes: maximal tumor blood flow/normal white matter, maximal tumor blood flow/normal gray matter, and maximal tumor blood flow/normal hemisphere. We used the maximal tumor blood flow/normal gray matter ratio for the assessments because the other two indexes were not available in the included studies. Our results indicated that the diagnostic accuracy was sufficiently high [32]. The DOR is a parameter that combines sensitivity and specificity, and ranges from zero to infinity; the higher the value, the better the discriminatory ability [33,34]. The pooled DOR was 92.47 (39.61–215.92), indicating that the overall accuracy was relatively high. The PLR and NLR represent the diagnostic ability in clinical practice. A PLR > 10 and NLR < 0.1 denote high accuracy [35]. We found that the pooled PLR was 10.40 and the pooled NLR was 0.11, indicating high clinical diagnostic ability.

The major strength of this meta-analysis was as follows: first, we realized that the heterogeneity may be high amongst the studies. That is why we first conducted a threshold effect test. The Spearman’s test indicated that there is no threshold effect within studies (*r* = 0.496, *P*=0.175), which means the heterogeneity with studies is not induced by the threshold effect. Moreover, the heterogeneity for sensitivity was 0.00%, and the specificity showed a moderate heterogeneity (49.1%). This is under the range of control. We used the random-effects model to conduct such combination. We conducted sensitivity analyses through sequentially excluding certain studies, and the summary sensitivity and specificity, PLR, NLR, and SROC were altered, indicating that the present pools estimated were stable. We followed the PRISMA guidelines and the recommendations of the Cochrane collaboration. However, our meta-analysis also had some limitations. First, we searched only four online databases and some unpublished data may exist. Second, the included studies had small sample sizes, although a meta-analysis is more powerful than an individual study. Third, other indexes of arterial spin labeling were not evaluated, such as the maximal tumor blood flow/normal gray matter and maximal tumor blood flow/normal hemisphere ratios, because these data were not present in the included studies. Finally, the included studies were of Asian populations, and other populations should be examined.

In conclusion, the normalized maximal tumor blood flow/normal white matter ratio determined using arterial spin labeling is relatively accurate for distinguishing high- from low-grade glioma. As a non-invasive procedure with favorable repeatability, this index may prove useful in clinical diagnostics.

## Supporting information

## Abbreviations

AUC: area under the curve
CI: confidence interval
CT: computed tomography
DCE-MRI: dynamic contrast-enhanced MRI
DOR: diagnostic odds ratio
DSC-PWI: dynamic susceptibility contrast perfusion-weighted imaging
NLR: negative likelihood ratio
nTBF: normalized tumor blood flow
PASL: pulsed arterial spin labeling
PLR: positive likelihood ratio
PWI: perfusion-weighted imaging
QUADAS-2: Quality Assessment of Diagnostic Accuracy Studies 2
SROC: summary receiver operating characteristic
