# Supplementary material for: Clinical utility of arterial spin labeling for preoperative grading of glioma
Source: Biosci Rep. 2018 Aug 31;38(4):BSR20180507. doi: 10.1042/BSR20180507 (PMC6117615; doi:10.1042/BSR20180507)
Supplement: Supplementary file 1 [file bsr20180507_Supp1.pdf]

Search Strategy

PubMed (searched on April 30, 2018)

Year (from inception to 2017)

Step 1: “glioma”/ all subheading [MeSH] OR “glial tumor”/all subheadings [all fields] OR “malignant glioma”/ all subheadings [all fields] OR “HGG” /all subheadings [all fields] OR “Brain neoplasms” /all subheadings [all fields]

Step 2: “arterial spin-labeling”/all subheadings [all fields] OR “ASL perfusion”/all subheadings [all fields] OR “Magnetic resonance imaging” /all subheadings [all fields] OR “Magnetic resonance angiography” /all subheadings [all fields] AND results from Step 1

Step 3: ‘‘diagnostic test”/all subheadings [ail fields] OR " diagnostic ability”/ all subheadings [all fields] AND results from Step 2

Web of Knowledge and Embase (searched on April 30, 2018)

Year (from inception to 2017)

Step 1: “glioma”/ [Topic] OR “glial tumor, / [Topic] OR ‘‘Brain neoplasms, / [Topic]

Step 2; " Magnetic resonance imaging " / [Topic] OR “Magnetic resonance angiography”/ [Topic] OR “arterial spin-labeling” / [Topic] / “ASL perfusion” / [Topic] / “Magnetic resonance imaging” / [Topic] /

AND results from Step 1

中文检索策略

中国知网、万方数据库和维普数据库

(检索日期：2018 年 4 月 30 日)

年限（建库至 2018 年）

步骤 1：“胶质瘤”/[主题词]OR“脑肿瘤”

步骤 2：在步骤 1 的基础上“磁共振成像”/[主题词]OR“磁共振血管造影术”/[主题词]OR “动脉自旋标记技术”/[关键词]

步骤 3:在步骤 2 的基础上加上“诊断” OR“分级”

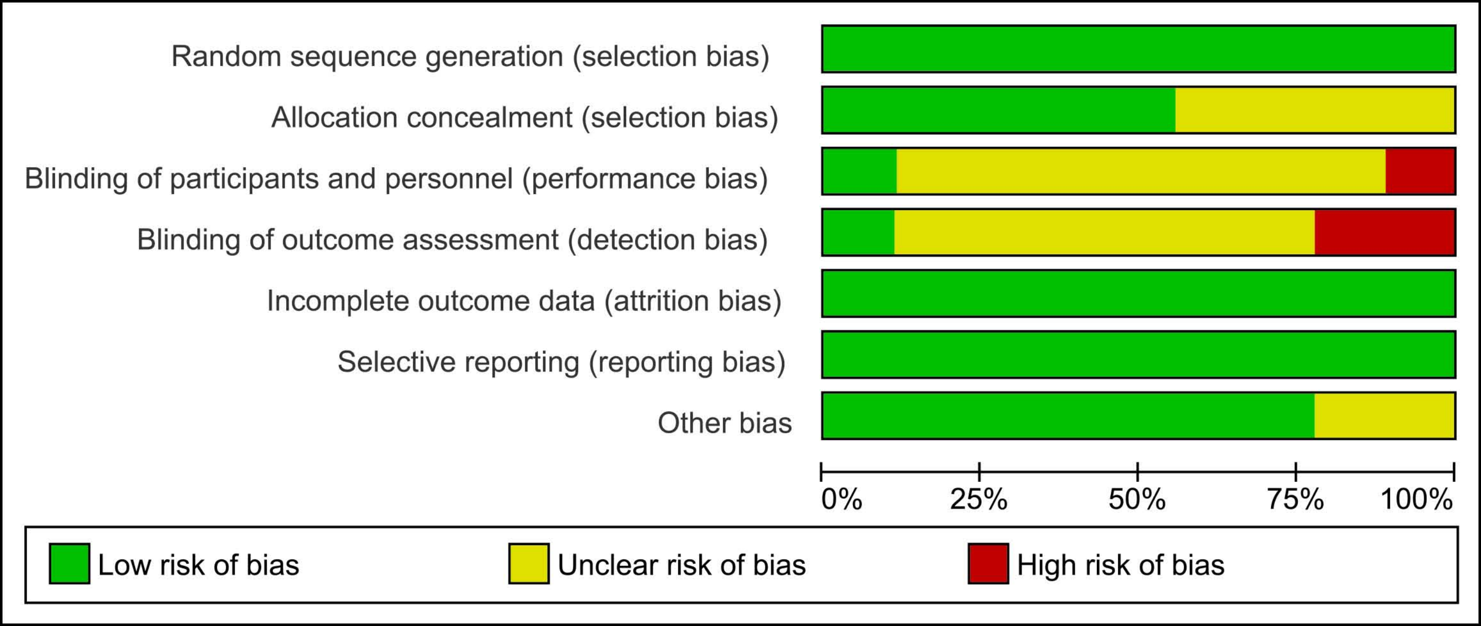
Random sequence generation (selection bias)
Allocation concealment (selection bias)
Blinding of participants and personnel (performance bias)
Blinding of outcome assessment (detection bias)
Incomplete outcome data (attrition bias)
Selective reporting (reporting bias)
Other bias
0%
25%
50%
75%
100%
Low risk of bias
Unclear risk of bias
High risk of bias

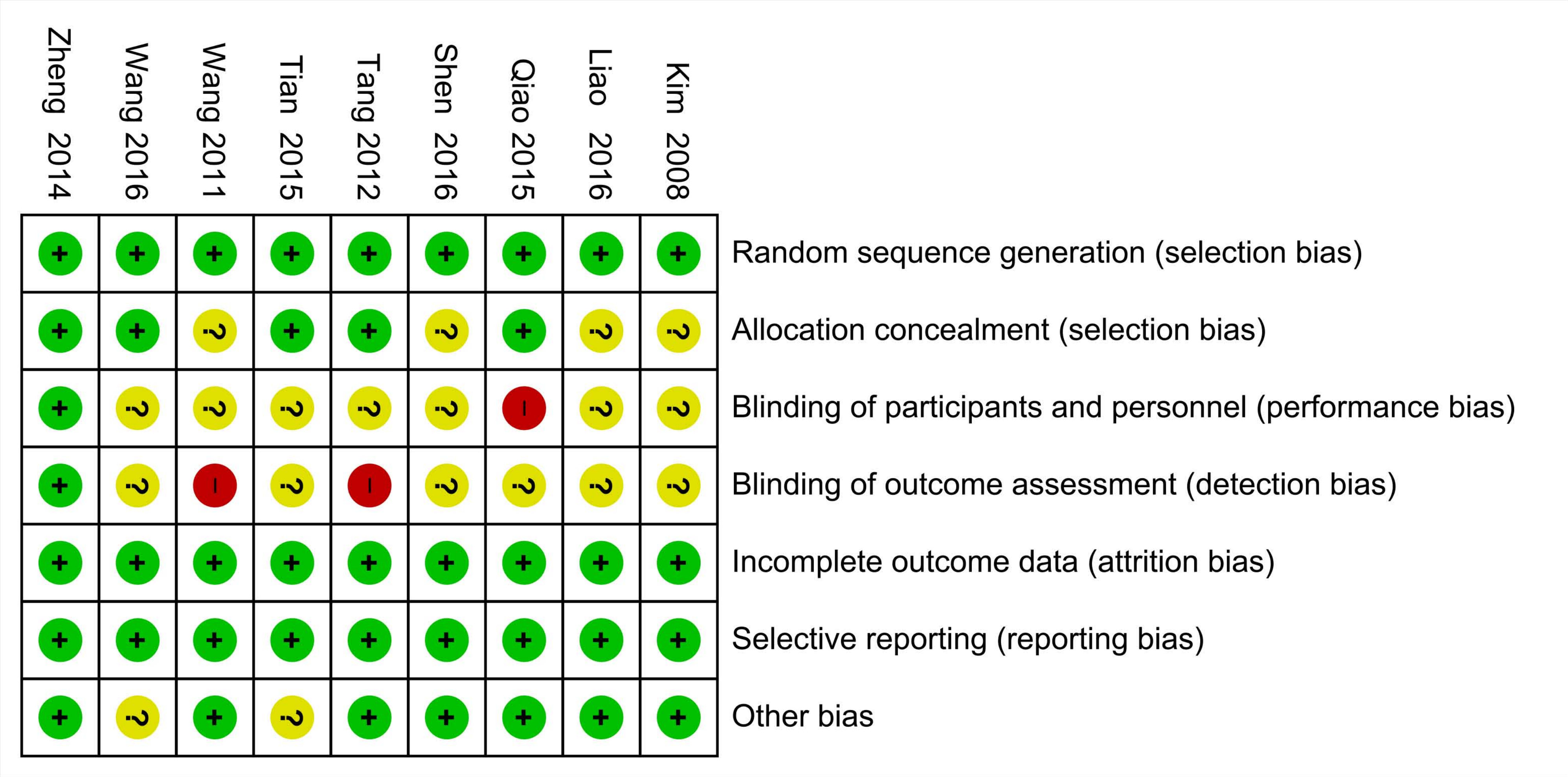

Zheng 2014
Wang 2016
Wang 2011
Tian 2015
Tang 2012
Shen 2016
Qiao 2015
Liao 2016
Kim 2008
Random sequence generation (selection bias)
Allocation concealment (selection bias)
Blinding of participants and personnel (performance bias)
Blinding of outcome assessment (detection bias)
Incomplete outcome data (attrition bias)
Selective reporting (reporting bias)
Other bias
